# Acute and Persistent *Mycobacterium tuberculosis* Infections Depend on the Thiol Peroxidase TPX

**DOI:** 10.1371/journal.pone.0005150

**Published:** 2009-04-02

**Authors:** Yanmin Hu, Anthony R. M. Coates

**Affiliations:** Centre of Infection, Division of Cellular and Molecular Medicine, St George's University of London, London, United Kingdom; Instituto Butantan, Brazil

## Abstract

The macrophage is the natural niche of Mycobacterium tuberculosis infection. In order to combat oxidative and nitrosative stresses and persist in macrophages successfully, M. tuberculosis is endowed with a very efficient antioxidant complex. Amongst these antioxidant enzymes, TpX is the only one in M. tuberculosis with sequence homology to thiol peroxidase. Previous reports have demonstrated that the M. tuberculosis TpX protein functions as a peroxidase in vitro. It is the dominant antioxidant which protects M. tuberculosis against oxidative and nitrosative stresses. The level of the protein increases in oxidative stress. To determine the roles of tpx gene in M. tuberculosis survival and virulence in vivo, we constructed an M. tuberculosis strain lacking the gene. The characteristics of the mutant were examined in an in vitro stationary phase model, in response to stresses; in murine bone marrow derived macrophages and in an acute and an immune resistant model of murine tuberculosis. The tpx mutant became sensitive to H_2_O_2_ and NO compared to the wild type strain. Enzymatic analysis using bacterial extracts from the WT and the tpx mutant demonstrated that the mutant contains reduced peroxidase activity. As a result of this, the mutant failed to grow and survive in macrophages. The growth deficiency in macrophages became more pronounced after interferon-γ activation. In contrast, its growth was significantly restored in the macrophages of inducible nitric oxide synthase (iNOS or NOS2) knockout mice. Moreover, the tpx mutant was impaired in its ability to initiate an acute infection and to maintain a persistent infection. Its virulence was attenuated. Our results demonstrated that tpx is required for M. tuberculosis to deal with oxidative and nitrosative stresses, to survive in macrophages and to establish acute and persistent infections in animal tuberculosis models.

## Introduction

*Mycobacterium tuberculosis* lives and survives in macrophages which generate antimicrobial radicals such as reactive oxygen species (ROS) and reactive nitrogen species (RNS) especially after activation with interferon gamma [1]. ROSs are produced by the macrophage NADPH oxidase (Phox) which reacts with molecular oxygen to form superoxide (O_2_^−^). O_2_^−^ then can be converted to the toxic H_2_O_2_ and the hydroxyl radical [2]. The RNSs are generated by the inducible nitric oxide synthase (NOS2). Also NO and superoxide can react to form highly toxic products such as peroxynitrite [3], [4]. As a successful intracellular pathogen, *M. tuberculosis* has evolved with powerful defence strategies to detoxify superoxide and nitric oxide [5] in order to maintain its viability and to achieve long-term persistence in human organs. The mechanisms by which *M. tuberculosis* detoxifies ROI and RNI are of particular interest because this knowledge will help us to understand the organism's pathogenesis and its ability to persist, which in turn leads to latent infection. As in other bacteria, *M. tuberculosis* has an array of enzymes with 22 detoxification genes [6] encoding catalases, peroxidases [7], [8], [9] and superoxide dismutases [10], [11] which may have roles in the detoxification and metabolism of reactive oxygen and nitrogen species. *M. tuberculosis katG* encodes an enzyme which exhibits both catalase, peroxidase and peroxynitritase activities which detoxify and metabolise the reactive oxygen and nitrogen species [12], [13], [14], [15]. The peroxiredoxin-type peroxidases, alkyl hydroperoxide reductase (AhpC) and thioredoxin peroxidase (TpX) have important antioxidant protection of this organism against oxidative and nitrosative stresses [9], [16], [17], [18], [19]. Also KatG and AhpC mediate resistance of *M. tuberculosis* to isoniazid [16]. *M. tuberculosis* contains only one *tpx* (Rv1932) encoding a putative thiol peroxidase [6]. The TpX has been characterised as a single cysteine peroxiredoxin with cys_60_ which serves as a reaction site for either oxidising or reducing substrates [20]. The activity of the *M. tuberculosis* TpX with thioredoxin as an electron donor in the reaction with hydroperoxides and peroxynitrite is significantly greater than that of AhpC [21] suggesting that TpX is the most efficient enzyme to provide protection of *M. tuberculosis* against oxidative and nitrosative stress. Also multiple thioredoxin-mediated pathways play important roles in the detoxification of hydroperoxides in *M. tuberculosis* [9], [21]. Although the physical property and structure of *M. tuberculosis* TpX as a peroxiredoxin have been studied *in vitro* [20], [21], [22], [23], its role in *M. tuberculosis* virulence and pathogenesis has not been explored. In *Escherichia coli*, a *tpx* deleted mutant grew slower than the WT strain and became sensitive to oxidative-stress [24], indicating that the TpX protein functions in vivo as a peroxidase. Expression of a yeast thioredoxin peroxidase renders *E. coli* resistant to oxidative stress induced by singlet oxygen [25]. In *Enterococcus faecalis,* analysis of mutants lacking three peroxidases including a NADH peroxidase, an Alkyl hydroperoxide reductase and a thiol peroxidase (*tpx*), respectively or a triple mutant revealed that TpX showed the most significant antioxidant activity to protect the bacterium to survive in macrophages. Also the *tpx* mutant was attenuated in a mouse peritonitis model [26].

To determine the roles of *tpx* gene in *M. tuberculosis* survival and virulence in vivo, we generated a *tpx* deleted mutant. The characteristics of the mutant were examined in an in *vitro* stationary phase model [27], in response to stresses, in murine bone marrow derived macrophages and in an acute and an immune resistant model of murine tuberculosis [28]. We demonstrated that the *tpx* mutant is sensitive to the reactive oxygen and nitrogen species. The mutant failed to survive in macrophages and in murine tuberculosis models.

## Materials and Methods

### Bacterial strains and growth conditions

*Escherichia coli* XL1 was used as a host strain for cloning and plasmid propagation. *E. coli* strains were cultured in or on liquid and solid Luria-Bertani medium. *M. tuberculosis* strain H37Rv was used as the wild type strain (WT) to construct the *tpx* mutant. All *M. tuberculosis* strains were grown in 7H9 medium containing 0.05% Tween 80 supplemented with 10% albumin dextrose complex (ADC; BD) without disturbance for the desired period of time, or on 7H11 agar medium supplemented with oleic albumin dextrose complex (OADC, BD). Antibiotics were used as follows: ampicillin (Sigma) 100 µg/ml, kanamycin (Sigma) 25 µg/ml, gentamicin (Sigma) 20 µg/ml and hygromycin (Invitrogen) 100 µg/ml.

### DNA manipulations, PCR and sequencing

All DNA techniques including DNA isolation, ethanol precipitation of DNA, electrophoresis of DNA in agarose and transformation were performed by standard techniques [29]. Enzyme reactions were performed according to the manufacturer's instructions (Invitrogen and New England Biolabs). Plasmids were extracted using a QIAprep Spin Miniprep Kit (Qiagen). PCR was performed in a total volume of 50 µl containing 200 µM each of dATP, dCTP, dGTP and dTTP, 1 µM of each primer, 10 ng of DNA, 1 unit of Hotstar Taq polymerase (Qiagen) and buffer system supplied with the enzyme. The PCR was amplified for 30 cycles (94°C 1 min, 58°C, 2 min and 72°C 3 min), followed by a final extension of 10 min at 72°C. PCR products were purified from agarose gel using a QIAquick Gel Extraction Kit (Qiagen). Both DNA strands were sequenced commercially (Qiagen, German) using the two primers that were used to generate each PCR product.

### Construction of an *M. tuberculosis tpx* mutant

The *tpx* deleted construct was made using the 2-step mutagenesis strategy as described previously [30]. A PCR product containing the *tpx* gene (498 bp) and about 1 kb flanking sequences adjacent to each end of the gene was amplified using *M. tuberculosis* H37Rv genomic DNA as template and primers *tpx*1 (5′-AATAAGCTTCAGGTTTCGCAGCACCTCGT-3′) and *tpx*2 (5′-AATAAGCTTGCCCGAAGTGCTCTGCTGAC-3′). The PCR product was cloned into the *Hin*d III site of pGEM3Z (promega) to form pGEM*tpx*. The *tpX* gene was deleted by PCR with primers *tpx*M1 (5′-ATTACGCGTGGGCACAGTCTGCCAAGACC-3′) and *tpx*M2 (5′-ATTACGCGTGCCGCGCTGGGCGCCTAGGC-3′) which were designed outwardly starting from the start and stop codons of the *tpx* gene using pGEM*tpx* as a template. The PCR product which contains only the flanking sequences of the *tpx* gene was cut with MulI and ligated to form pGEMΔ*tpx*. The flanking sequences were cloned into the *Hind* III site of p2NIL [30] to make p2NILΔ*tpx*. A hyg -sacB marker cassette from the pGOAL 19 [30] was cloned into the *Pac* I site of p2NILΔ*tpx* to form the final mutant construct p2NILΔ*tpx*1 which was electroporated into *M. tuberculosis* H37Rv cells. The selection of the *tpx* mutant was performed as described previously [31]. PCR screening of the *tpx* mutant was performed using the primers *tpx*CD1 (5′-CCGTCGGTGAGCTACCTGCT- 3′) and *tpx*CD2 (5′ -TGCGCGATTTCCGGCACCA- 3′) which were designed in the coding region of the *tpx* gene.

To complement the *tpx* deletion, a 992 bp DNA fragment containing the *tpx* gene and 377 bp of upstream sequence was amplified by PCR using primers *tpx*C1 (5′-AATGAATTCCCGGAAATTCGCGGGCGAA-3′) and *tpx*C2 (5′-AATGAATTCTCCACGGTGCCATCGCCTT-3′). The PCR product was cloned into the *EcoR I* sites of the integrating plasmid pYH10 [32]. The construct was transformed into *tpx* mutant by electroporation and gentamicin resistant transformants were selected.

### Estimation of viability under stress conditions

*M. tuberculosis* strains were grown in 7H9 medium containing 0.05% Tween 80 supplemented with 10% ADC without disturbance for 7 to 10 days. A series of 10 ml standing cultures were used for the determination of CFU counts after exposure to stresses. For oxidative stress, H_2_O_2_ (Sigma) or paraquat (Sigma) was added to the cultures at the final concentration of 5 and 10 mM for H_2_O_2_ and 10 and 20 mM for paraquat. For nitric oxide stress, diethylenetriamine/nitric oxide adduct (DETA/NO, Sigma) or S-nitrosoglutathione (GSNO, Sigma) was added to the cultures at 1.25, 2.5 and 5 mM for DETA/NO and 5 and 10 mM for GSNO, and then the cultures were incubated at 37°C for 24 hours. CFU counts of the treated cultures and the non-treated cultures were determined. Each stress treatment was carried out in duplicate.

### Mouse infection models

Female BALB/c mice (6–8 weeks old) were obtained from Harlan, UK Ltd. *M. tuberculosis* strains which were grown to mid-log phase at 10 days were resusupended in phosphate-buffered saline (PBS). The mice were intravenously infected with 10^5^ CFU bacterial cells/mouse. At various time points, spleens and lungs from 4 mice were removed rapidly after sacrifice and a sterile autopsy was performed. The organs were transferred into 2 ml tubes each containing 1 ml sterile distilled water and 2 mm diameter glass beads. Lungs and spleens of the mice were homogenised using a FastPrep Instrument (Fisher Scientific) for 40 seconds at 6.5 speed. CFU counts of the organs were performed using the diluted homogenates.

Virulent assay of the *M. tuberculosis* strains with SCID mice (6–8 week, female, Harlan UK Ltd) were carried out as described previously [33]. Three groups of mice (each contains 9 mice) were intravenously infected with 10^6^ CFU of the WT, mutant and the complemented strains which were grown to mid-log phase. After 4 hours of infection, three mice in each group were sacrificed and CFU counts in lungs and spleens were estimated. The remaining 6 infected mice were observed for 60 days and the death time for each group was recorded. Median survival times were calculated for each group, and statistical analysis was carried out using the log rank tests of survival (GraphPad Prim Software).

The animal husbandry guidelines for this study were followed according to the Animals Scientific Procedures Act, 1986 (an Act of the Parliament of the United Kingdom 1986 c. 14).

### Macrophage infection

Bone marrow derived macrophages were cultured as described previously [33], [34]. Bone marrow cells were flushed from the femurs of BALB/c, C57BL/6 and iNOS knockout mice (C57BL/6 iNOS^−/−^) (6–8 week old) and cultured in Dulbecco's Modified Eagle Medium (Invitrogen) supplemented with 10% Foetal Bovine Serum (Invitrogen), 20% L-cell conditioned medium, 4500 mg/L of D-glucose, 4 mM of L-glutamine. 110 mg/L of sodium pyruvate, 100 U/ml of penicillin and 100 µg/ml of streptomycin at 37°C, 5% CO_2_ for 7 days. Adherent macrophages were washed 3 times with warm Hanks' buffered salt solution (HBSS, Sigma). The macrophages were harvested and seeded at 1.25×10^5^ cells per well in the 24 well plates in the above culture medium without L-cell conditioned medium and antibiotics. Activation of the bone marrow derived macrophages was carried out by the addition of γ-interferon (IFN-γ, 100 U/ml. R&D Systems) for 24 hours and followed by the addition of lipopolysaccharide (LPS, 200 ng/ml, Sigma) for 3 hours. The cells were infected with the WT, *tpX* mutant and the complemented strain at a multiplicity of infection of 1∶1 for 4 hours, and then the cells were washed 5 times with warm HBSS. At day 0, 2, 4 and 6 days after infection, the cells were washed and lysed with 0.1% Triton-X 100. CFU counts were performed at appropriate dilutions of the lysed cell suspension. At each time point, the infected macrophages in two wells were individually harvested using Trypsin- EDTA and stained for acid-fast bacilli to check if the bacilli were maintained inside macrophage in order to verify macrophage viability. The infection experiments were carried out in duplicate for three times.

### Measurement of peroxidase activity in WT, Δ*tpx* and the complemented strains

Peroxidase activity was determined using the cell lysates of the WT, *tpx* mutant and the complemented strains with H_2_O_2_ as a substrate. The decomposition of H_2_O_2_ was measured using xylenol orange assay which detects peroxide based on oxidation of ferrous to ferric ion in the presence of xylenol orange and provides a sensitive colorimetric measurement spectrophotometrically at 560 nm. PeroXOquant™ Quantitative Peroxide Assay Kit (Pierce) was used. *M. tuberculosis* strains were grown in 7H9 medium containing 0.05% Tween 80 supplemented with 10% ADC without disturbance for 10 days. Bacterial numbers of the cultures were determined by optical density reading at 600 nm and CFU counts. The cultures were washed three times with water. The cell pellets were transferred into 2 ml tubes each containing 1 ml sterile distilled water and 75 to 150 µm glass beads and lysed by homogenisation using a FastPrep Instrument (Fisher Scientific) for 40 seconds at 6.5 speed. The cell debris was removed by centrifugation at 13,000 rpm for 20 minutes followed by filtration. Total protein concentrations of the cell lysates were determined with the Bio-Rad protein assay using BSA as a standard. 20 µl of reaction mixture containing H_2_O_2_ and the cell homogenate was added to 200 µl of working reagent of PeroXOquant™ kit and then incubated at room temperature for 30 min. The remaining amount of H_2_O_2_ was determined by measuring the absorbance at 560 nm. The cell homogenate containing the same amount of total protein derived from 10^7^ bacterial cells from WT, *tpx* mutant and the complemented strain was used for all reactions.

### Statistical analysis

The difference between different experimental groups was determined by Student T test. P value <0.05 is considered significant.

## Results

### *M. tuberculosis tpx* mutant construction

**Figure 1:**
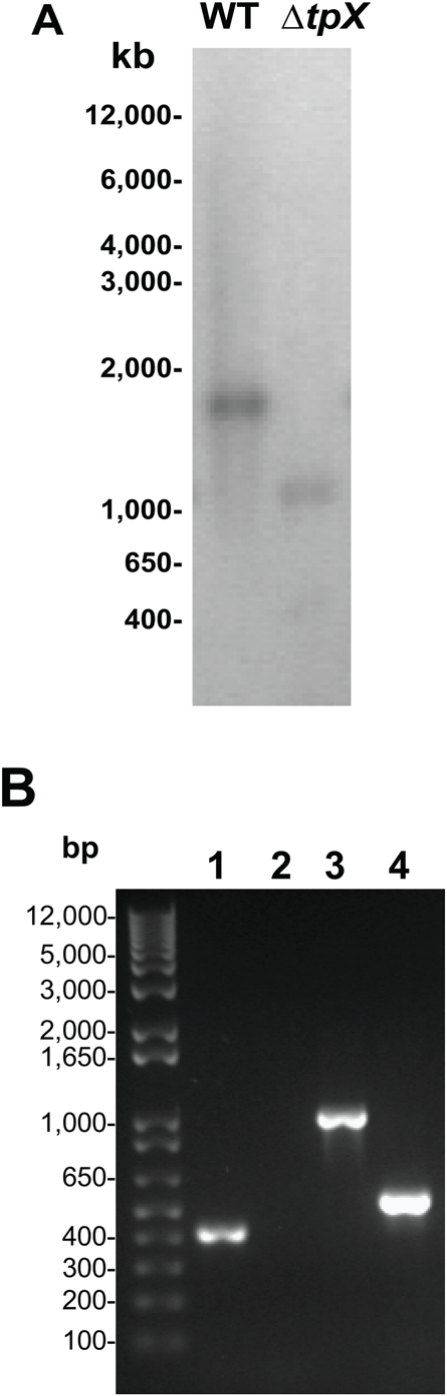
Confirmation of the *M. tuberculosis tpx* gene deletion. A. Southern blotting analysis of DNA from the WT and the YHΔ*tpx.* which was digested with EcoRV and PvuI and hybridized with a probe synthesized to make the complemented construct. B. PCR amplification of DNA from the WT and the YHΔ*tpx.* 1. WT, 2. *tpx* mutant. The primers used were designed in the coding region of the *tpx* gene. 3. WT, 4. *tpx* mutant. The primers used were for the amplification of complemented construct. M, molecular weight marker (Invitrogen). The experiments were repeated twice, with identical results.

Construction of the *tpx* mutant was performed using a two-step mutagenesis strategy [30]. Complete in-frame deletion of the *tpx* gene was carried out by PCR using the method as described previously [34]. The generation of the mutant strain was confirmed by Southern blotting analysis (Fig. 1A) and PCR (Fig. 1B) in which no PCR product was amplified in the mutant using the primers *tpx*CD1 and *tpx*CD2 which were designed in the coding region of the gene. Both sequences of the PCR product amplified from the mutant were analysed by DNA sequencing to confirm the deletion of the *tpx* gene (data not shown). The *tpx* deleted *M. tuberculosis* H37Rv strain was termed YHΔ*tpx*.

In order to complement the deletion of *tpx* gene, a 992 kb DNA fragment which contains the *tpx* coding region frame and the 377 bp upstream sequence of the gene was cloned into plasmid pYH10. The construct was transformed into the YHΔ*tpx* followed by selection for gentamicin resistance. The success of the complementation was confirmed by PCR (data not shown) and the strain was termed as **YH** ***tpx*** **Comp.**

The *tpx* gene contributes to survival under oxidative and nitrosative stress conditions *in vitro.*

The WT, YHΔ*tpx* and YH*tpx*Comp *M. tuberculosis* H37Rv strains were grown in 7H9 broth without disturbance for 100 days as described previously [35]. The viability of these strains was determined by CFU counts at different time points. Growth characteristics of the mutant were similar to those of the wild-type strain (data not shown), indicating that the *tpx* product is not required for the bacterium to grow *in vitro*.

**Figure 2:**
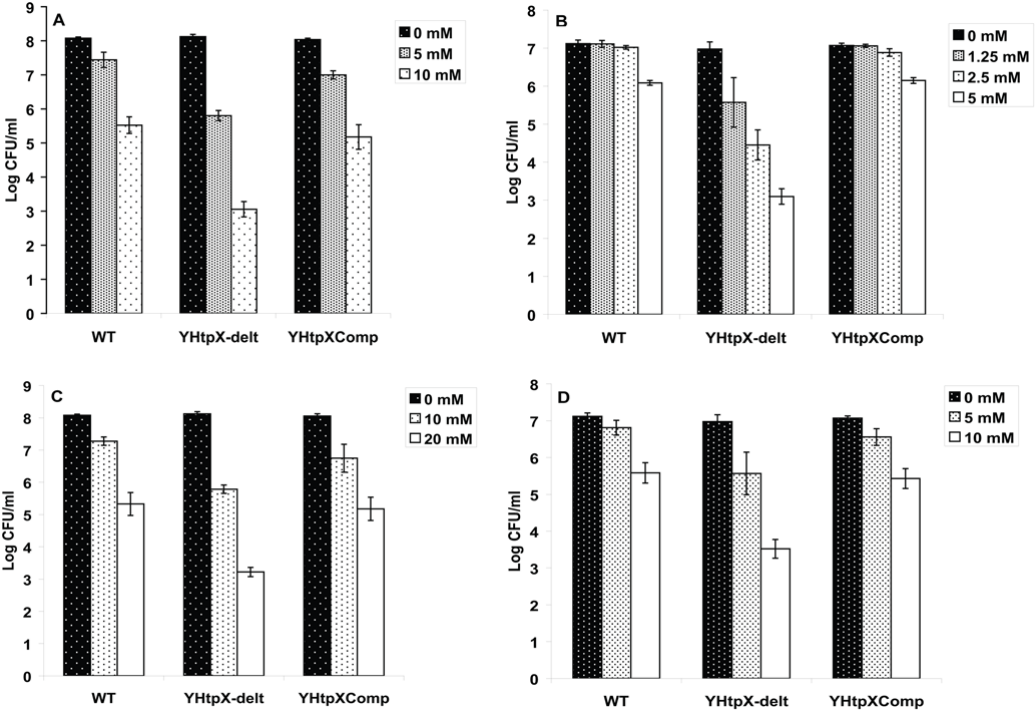
Inactivation of *tpx* gene renders the mutant more susceptible to oxidative and nitrosative stresses. Survival of YH*Δtpx* compared with WT and the complemented strains in response to H_2_O_2_ at 5 and 10 mM (A), DETA/NO at 1.25, 2.5 and 5 mM (B), paraquat at 10 and 20 mM (C) and GSNO at 5 and 10 mM (D). The data shown is a representative of three independent experiments. Data are represented as mean±SD of triplicate tests. The CFU counts in the Δ*tpx* are significantly lower using a t-test than in WT after exposure to both H_2_O_2_ and NO for 24 hours (p<0.0001 all concentrations for both stress conditions).

In order to determine the contribution of the *tpx* gene in the response of *M. tuberculosis* to oxidative and nitrosative stresses, the following experiment was performed: the WT, YHΔ*tpx* and YH*tpx*Comp were treated with H_2_O_2_ and DETA/NO which is a slow-release NO donor with a long half-life of about 20 hours for the liberation of NO [36]. Comparison of the survival rate of the mutant with the WT strain revealed significant differences in response to the stress conditions. As shown in Fig. 2A, after H_2_O_2_ treatment for 24 hours, viability of the strains decreased. However, compared with the WT strain, the *tpx* mutant became more sensitive to H_2_O_2_. At 5 mM and 10 mM of H_2_O_2_, there was a reduction of 0.64 and 2.55 logs in CFU counts for the WT strain whereas the reduction was 2.25 and 5 logs for the mutant (P<0.0001, H_2_O_2_ at 5 mM and 10 mM, respectively). A similar pattern was seen after DETA/NO treatment (Fig. 2B). At 1.25 and 2.5 mM of DETA/NO, there was no decrease in CFU for the WT strain but 1.4 and 2.52 log kill in the mutant, respectively. At 5 mM, there was 3.87 log decreases in the mutant in contrast to 1 log kill in the WT. The difference in reduction of CFU counts is significant between the WT strain and the mutant (P<0.0001 NO at 1.25, 2.5 and 5 mM. determined by student's T test, n = 3). The reduced tolerance to H_2_O_2_ and NO of the mutant was recovered in the complemented strain. The survival of the mutant under these stress conditions was also examined using paraquat and GSNO, a NO donor, and similar results to H_2_O_2_ and DETA/NO were observed (Fig. 2C and 2D). These results suggest that the *tpx* gene product plays important roles in the oxidative and nitrosative stress survival of *M. tuberculosis.*

### The *tpx* gene contributes virulence in mice

**Figure 3:**
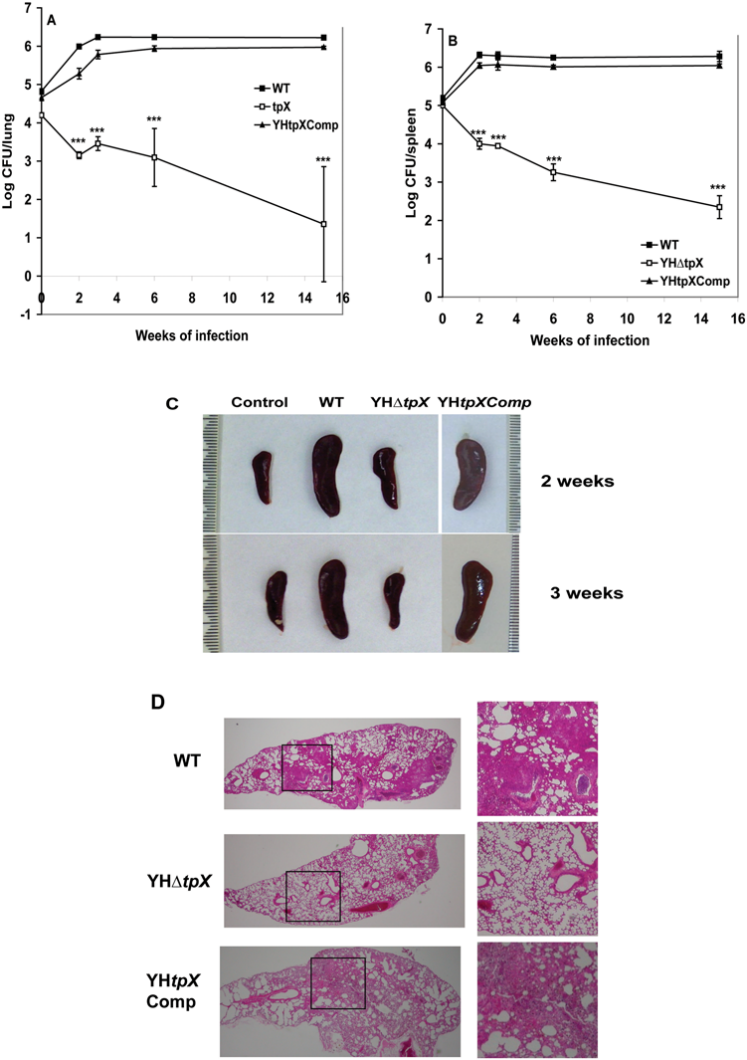
Growth and survival of YH*Δtpx in* the lungs and spleen of BALBc mice. Mice were infected with 3 × 10^5^ bacteria. At different time points the infected mice were sacrificed and the numbers of bacteria in the lung (A) or spleen (B) were measured. The results for each time point are the means and SDs of four mice in each experimental group. The experiments have been reproductively repeated twice with similar CFU counts in lungs and spleens. Statistical significance was determined by Student's *t* test (***, P<0.0001). C. Changes in spleen gross anatomy after infection with the WT, the mutant and the complemented strains. Spleens were collected at 2 and 3 weeks after infection. D. Lung histology of mice infected with the WT, the mutant and the complemented strains. Histopathological examination was performed using three mice in each group. Three sections from each mouse were examined. The images shown are representative of lung sections from three animals in each experimental group. Enlarged images of the boxed regions on the left panel (magnification, 4×) are shown on the right panel (magnification, 10×).

In order to examine if the *tpx* mutant is able to grow and persist *in vivo*, a medium-dose i.v. mouse infection model [34] was used in which the mice possess an immune response that restricts bacterial growth after two to three weeks of infection but fail to eliminate the bacilli. The WT, YHΔ*tpx* and YH*tpx*Comp were intravenously injected into the BALB/c mice with 10^5^ CFU of bacilli per mouse. The viability of the bacterial strains was determined by CFU counts for 15 weeks. As shown in Fig. 3, the CFU counts of the WT strain reached a peak at three weeks of infection in the mouse lungs (Fig. 3A) and at two weeks of infection in spleens with log 6.3 CFU/organ (Fig. 3B), then the bacterial numbers remained relatively constant up to 15 weeks of infection. However, the YHΔ*tpx* failed to grow in both lungs and spleens after initial infection. At 15 weeks, there were 1.35 log and 2.35 log remaining in the lung and spleen, respectively. The parental levels of the CFU counts from lungs and spleens were recovered in the complemented strain. There were significant differences in CFU counts between the WT and the mutant infected organs (p<0.0001).

To examine if the mice infected with the *tpx* mutant produced more inflammatory response than those infected with the WT strain, which inhibited the growth of the mutant in the mouse organs, the spleen gross anatomy was observed. As shown in Fig. 3C, after two and three weeks of infection, enlarged spleen sizes were seen in the WT strain infected mice. In contrast, the *tpx* mutant infected spleens were of similar size to those of the non-infected mice, indicating that the *tpx* mutant failed to induce the host's inflammatory response against the bacterial infection.

Histopathological examination of the lungs infected with the mutant and the WT strains demonstrated a marked difference. As shown in Fig. 3D, at 15 weeks of infection, there are large areas of granulomatous inflammation with increased numbers of inflammatory cells including lymphoplasmacytic cells, macrophages, neutrophils and multinucleated cells in the WT strain infected lungs, but in the mutant infected lungs, only very occasional inflammatory cells are present in the alveolar walls with normal alveoli and airways evident throughout the lungs.

**Figure 4:**
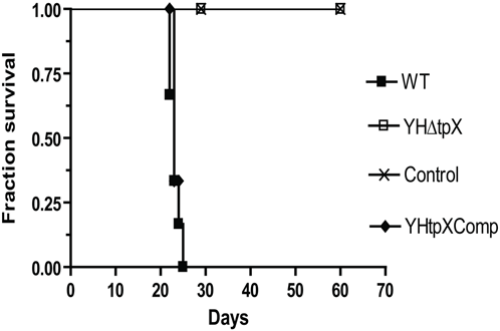
Survival of SCID mice (*n* = 6 per group) infected intravenously with *M. tuberculosis* H37Rv WT, YHΔ*tpx* and the complemented strains. Deletion of *tpx* gene led to no death of the mice over 60 days after infection.

In order to examine if the mutant was able to establish an acute infection, we infected SCID mice with the WT, the mutant and the complemented strains. Median survival times for the mice were observed over 60 days after infection. Similar CFU counts of the WT, YHΔ*tpx* and YH*tpx*Comp were recovered from mouse lungs and spleens after 4 hours of infection (CFU counts: WT, 5.88 logCFU/lung and 6.02 logCFU/spleen. YHΔ*tpX,* 5.87 logCFU/lung and 6.01 logCFU/spleen. YH*tpX*Comp, 5.87 logCFU/lung and 5.99 logCFU/spleen). As shown in Fig. 4, the median survival time of the WT strain infected mice was 23 days. However, the mice infected with the mutant remained healthy and no death was observed for 60 days. The parental level of virulence was significantly restored in the complemented strain (p<0.001).

### The *tpx* gene is essential for growth and survival in macrophages

**Figure 5:**
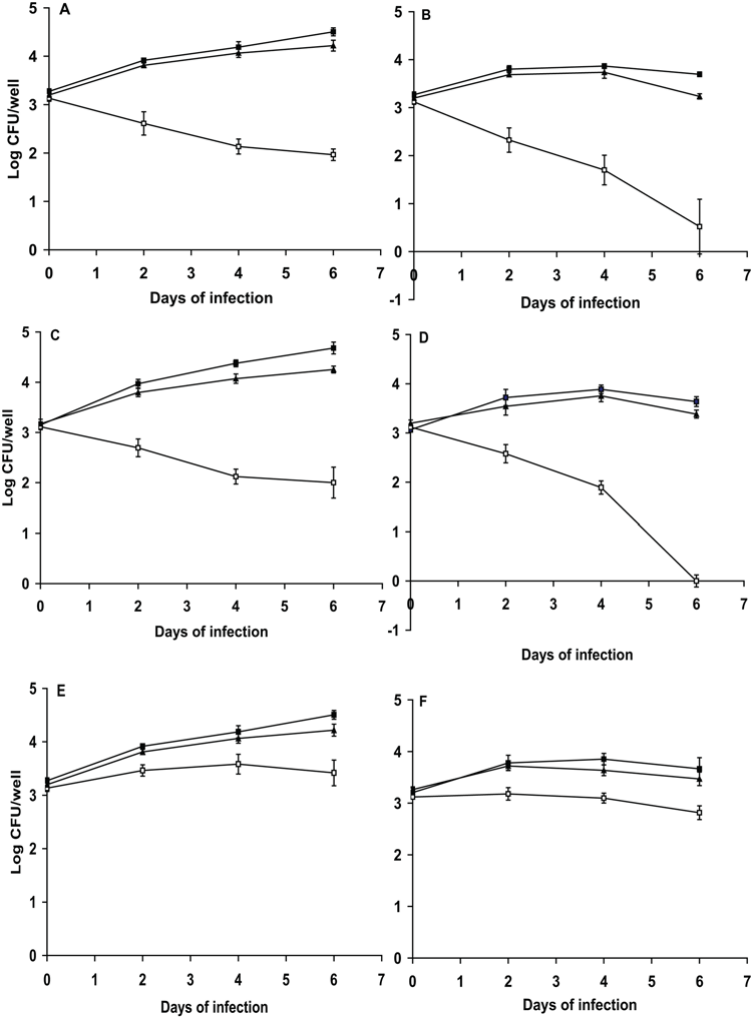
Growth and survival of *M. tuberculosis* Δ*tpx* in resting and IFN-γ-activated macrophages. A. Infection in resting bone marrow derived macrophages from BALB/c mice. B. Infection in IFNγ activated bone marrow derived macrophages from BALB/c mice. These results are the means and standard deviation derived from one representative of three independent experiments. C. Infection in resting bone marrow derived macrophages from C57BL/6 mice. D. Infection in IFNγ activated bone marrow derived macrophages from C57BL/6 mice. E. Infection in resting bone marrow derived macrophages from iNOS KO mice. F. Infection in IFNγ activated bone marrow derived macrophages from iNOS KO mice. The results are the means and SDs derived from triplicate wells. The experiments have been reproductively repeated once. Solid square: WT *M. tuberculosis* H37Rv. Open square: YHΔ*tpx.* Solid triangle, YH*tpx*Comp.

In order to investigate if the failure to grow in the mouse organs is due to the inability of the mutant to survive inside the macrophages, we examined the growth and survival of the mutant in resting and IFN-γ activated murine bone marrow derived macrophages which generate an oxidative burst by producing ROS and RNS [37]. As shown in Fig. 5A, in the resting macrophages the cell numbers of WT increased, but the CFU counts of the mutant decreased significantly after infection, and there is 2.54 log decrease of CFU counts in the mutant compared with the WT strain at 6 days of infection. In the activated macrophages, the reduction of CFU counts in the mutant was much more pronounced, at 6-days of post infection, 0.5 logs of the mutants cells were recovered (Fig. 5). In both resting and activated macrophages, the CFU counts recovered from the macrophages at time 0 were similar between the WT and the mutant, indicating that the mutant retained its ability to invade macrophages, but failed to grow and survive in them. The complemented strain showed a similar growth rate to the WT strain. These data suggest that *tpx* is essential for *M. tuberculosis* to overcome stresses and to survive in macrophages.

In order to verify that the failure of the mutant to survive in macrophages is due to the mutant's loss of its ability to detoxify reactive nitrogen species, we infected the iNOS KO mice and C57BL/6 (WT) mice with *M. tuberculosis* WT, YHΔ*tpx* and the complemented strains. As shown in Fig. 5C and 5D, in the macrophages of C57BL/6, the *tpx* mutant failed to survive in both resting and activated macrophages which is similar to the growth in the macrophages of BALB/c mice. However, in the iNOS KO macrophages, the growth of the *tpx* mutant was significantly restored. About 1 log kill of the mutant was seen in both activated and resting macrophage at 6 days of infection. In contrast, there are 3.64 and 2.68 log kill in macrophages of WT mice.

### The *tpx* mutant has reduced peroxidase activity

**Figure 6:**
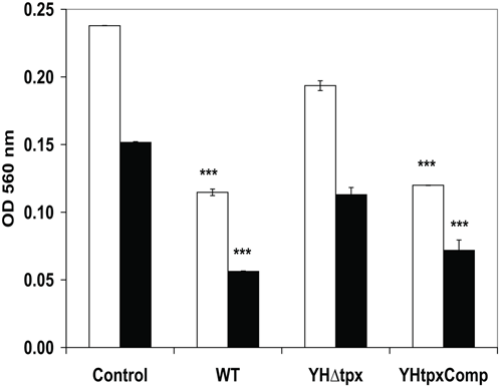
Biochemical analyses of peroxidase activities in *M. tuberculosis* WT, *tpx* mutant and the complemented strain. Presence of hydrogen peroxide was measured by xylenol orange assay. The open bar, initial H_2_O_2_ at 1 mM. The solid bar, initial H_2_O_2_ at 0.5 mM. Control, H_2_O_2_ only. The data was repeated twice with similar results.

In order to further investigate that the *tpx* mutant was unable to decompose peroxides, peroxidase activities of the WT, HYΔ*tpx* and HY*tpx*Comp were measured using xylenol orange assay. Equal amounts of total protein from 10^7^ bacterial cells were used in each reaction containing 1 mM and 0.5 mM of H_2_O_2_. The remaining H_2_O_2_ level was measured by comparing the difference between the test and the standard curve generated with H_2_O_2_. As seen in Fig. 6, the WT strain contained a high level of peroxidase activity and a significant reduction of H_2_O_2_ was seen. There are 0.32 mM and 0.18 mM of H_2_O_2_ remaining with initial addition of H_2_O_2_ concentration at 1 and 0.5 mM, respectively. In contrast, a low level of peroxidase activity was seen in the *tpx* mutant. There are 0.72 and 0.3 mM H_2_O_2_ remaining. There are significant differences in the enzyme activities between the WT and the mutant strains (p<0.0001). The peroxidase activity was restored in the complemented strain.

## Discussion

In this study we successfully generated an *M. tuberculosis* strain lacking the *tpx* gene. Our Δ*tpx* strain showed increased susceptibility to exogenously provided H_2_O_2_ and nitric oxide (Fig 2). The oxidative activity of *tpx* mutant was significantly reduced when measured using a biochemical assay with the cell extract compared to the WT strain (Fig. 6). This demonstrates that TpX's biological function is a thiol-dependent peroxidase. We clearly demonstrated that the *tpx* mutant failed to grow in macrophages of WT mice which are capable of generating an oxidative burst and this die-off was exaggerated after the macrophages were activated with interferon gamma (Fig. 5). In addition, the growth of the mutant was significantly restored in macrophages of iNOS knockout mice which produce a limited oxidative burst [2]. The growth phenotype of the *tpx* mutant in iNOS KO macrophage further demonstrated that the *tpx* mutant lacks the ability to decompose peroxides and nitric oxides. Further more, the mutant failed to initiate an acute infection in both immune deficient and immune competent mice and failed to maintain a persistent infection. This indicates that TpX is essential to protect *M. tuberculosis* against RNS and ROS.

How do these findings of the *tpx* mutant compare with those for the peroxiredoxin-type peroxidases, AhpC and other enzymes which also protect *M. tuberculosis* against oxidative and nitrosative stresses [9], [16], [17], [18], [19]? Deletion of *ahpC* (*ahpC*::Km^R^) in *M. tuberculosis* resulted in an increased sensitivity to peroxynitrite. This mutant failed to grow and survive in macrophages, and died more quickly in activated macrophages [19]. It has been shown that *M. tuberculosis ahpC* gene could complement the loss of the same gene in *Salmonella typhimurium* in which the mutant was hypersensitive to reactive nitrogen intermediates [17]. However, in another report, an *ahpC*::Km(r) mutant strain [38] showed no increased sensitivity to H_2_O_2_ but became sensitive to cumene hydroperoxide exposure which implies that it can only degrade organic hydroperoxides. This mutant showed no growth changes in mice compared to the WT strains. A previous report [15] showed that deletion of *katG* results in attenuation of the *M. tuberculosis* mutant in wild-type C57Bl/6 and NOS2(−/−) mice lacking nitric oxide synthase, but showed no growth deficiency in gp91(Phox−/−) mice which lack the gp91 subunit of NADPH oxidase. This indicates that KatG catabolizes the peroxides generated by the phagocyte NADPH oxidase. However, the deficiency of the *katG* mutant in C57Bl/6 mice was only observed during the periods of 2–4 week of post infection, no further decrease in viability was seen afterward suggesting that the *katG* gene is required for the bacterium to survive in mice for a certain period of time. Another *M. tuberculosis* mutant which produced a reduced level of SOD using antisense-*sodA* was sensitive to killing by hydrogen peroxide which led to attenuation of the mutant in mouse lungs and spleen [37]. A further *M. tuberculosis* mutant, which had a copper and zinc-cofactored Superoxide dismutase (*sodC*) deletion [10], was more susceptible to superoxides and hydrogen peroxide than its parental strain, but grew at the same rate in guinea pigs as the WT strain. This indicates that mycobacterial *sodC* is not essential for intracellular growth within macrophages and does not contribute to *M. tuberculosis* pathogenicity in the guinea-pig. However, in another separate report, a *sodC* knockout mutant of *M. tuberculosis* was sensitive to ROS and RNS killing *in vitro* and in macrophages [37], especially in activated macrophages. But significant killing of the mutant was only seen during the first 6 hours of infection, after which the viability of the mutant leveled off, indicating that there are other enzymes which compensate for the loss of *sodC*. Deletion of all these genes results in the loss of tolerance to either oxidative or nitrosative stress. It is difficult to compare the activities of these enzymes in response to stress conditions because these mutants were generated in different studies. In *Enterococcus faecalis,* analysis of three mutants [26] which lack NADH peroxidase, alkyl hydroperoxide reductase and thiol peroxidase revealed that the viability of the *tpx* mutant reduced the most in response to oxidative stress and growth in macrophages. This suggests that TpX is the most important antioxidant in the protection of the bacterium against stress conditions. Furthermore the *tpx* mutant was attenuated in a mouse peritonitis model [26], In addition, *in vitro* enzymatic analysis of *M. tuberculosis* TpX and AhpC with different hydroperoxides including peroxynitrite showed that the TpX was more efficient and faster in peroxynitrite reduction than AhpC [21]. Also the protein level of TpX in *M. tuberculosis* is considerably higher than that of AhpC [38], [39]. Unlike the *katG* mutant [15] and the *sodC* mutant [37], in our study, the increased kill of the *tpx* mutant in activated macrophages (Fig. 5) and in mice (Fig. 3 and 4) indicates there are no alternative antioxidant activities which are able to compensate for the loss of *tpx*. All these data suggest that *tpx* plays a dominant role *in vivo* to protect *M. tuberculosis* in its environment when challenged with oxidative and nitrosative stresses.

The growth deficiency of our *tpx* mutant in mice suggests that thiol peroxidase is essential for the bacterium to initiate growth and to persist in the face of host innate and acquired immunity. The *tpx* mutant failed to replicate and grow in the SCID mice (Fig. 4) which mount normal innate immune responses, including natural killer cells, macrophages and granulocytes but are immunodeficient in both B and T lymphocytes, and led to no death of the mice. Also in the immune competent mice, the mutant failed to replicate immediately after infection. All these data indicate that innate immune responses are sufficient to kill the mutant, as seen in the resting macrophages which produced limited oxidative burst [37]. The CFU counts of the *tpX* mutant continued to fall when the acquired immune response became established after 2 to 3 weeks of infection (Fig. 3A and 3B). The acquired immune response evidence by the granuloma formation is closely associated with increased expression of inducible nitric oxide synthase (NOS2) in granuloma macrophages and enhanced levels of nitrate/nitrite circulating in infected mice [40]. As a result of this, *M. tuberculosis* needs to constantly battle with ROS and RNS in order to establish a latent infection. The *tpx* mutant does not stimulate enhanced host immune responses, unlike the *sodA* mutant in which loss of virulence is due to an increased innate host immune response induced by the mutant itself [41]. An enlarged spleen is a marker for an increased level of host inflammatory response to bacterial infection [42], [43], [44], Splenomegaly develops in tuberculosis infection [34], [45]. In our experiments, during the first three weeks of infection, the spleen size of the WT strain infected mice was significantly enlarged (Fig. 3C). However, in the *tpx* mutant infected mice, the spleen size was the same as the non-infected control mice, indicating no increase in inflammation. Also the histopathological examination of the mouse lungs from both WT and the mutant infected mice revealed that most of the lungs of the mutant infected animals contain normal alveolar spaces and airways (Fig. 3D). These results demonstrate that the failure of the mutant to grow and persist in mouse organs is due to the mutant's inability to withstand oxidative and nitrosative stresses even in resting macrophages, and is not due to its induction of marked inflammatory responses.

In conclusion, our results show that *M. tuberculosis tpx* is an essential virulence factor. The *tpx* mutant is unable to establish acute and persistent infection. The major contribution of the *tpx* gene in *M. tuberculosis* pathogenesis depends on its antioxidant defense against oxidative and nitrosative stresses, which allows the bacterium to grow and survive in the macrophages.
